# The Nadir Oxygen-Specific Heart Rate Response in Sleep Apnea Links With the Occurrence of Acute Myocardial Infarction

**DOI:** 10.3389/fcvm.2022.807436

**Published:** 2022-04-26

**Authors:** Zhihua Huang, Yanpeng Wu, Kaizhuang Huang, Pingyan Chen, Jiyan Chen, Ling Wang

**Affiliations:** ^1^Department of Cardiology, Guangdong Cardiovascular Institute, Guangdong Provincial Key Laboratory of Coronary Heart Disease Prevention, Guangdong Provincial People’s Hospital, Guangdong Academy of Medical Sciences, Guangzhou, China; ^2^Department of Cardiology, Fuwai Hospital, National Clinical Research Center for Cardiovascular Diseases, National Center for Cardiovascular Diseases, Chinese Academy of Medical Sciences and Peking Union Medical College, Beijing, China; ^3^Department of Biostatistics, School of Public Health, Southern Medical University, Guangzhou, China; ^4^Department of Intensive Care Unit, The Fifth Affiliated Hospital of Sun Yat-sen University, Zhuhai, China

**Keywords:** sleep apnea, oxygen saturation, heart rate, myocardial infarction, cardiovascular risk

## Abstract

**Background:**

Little is known regarding the quantification of sleep apnea- and hypoxemia-elicited heart rate (HR) response and its prognostic significance of the cardiovascular risk. We sought to explore the impact of HR response and variability specific to obstructive sleep apnea (OSA) on the occurrence of a common cardiovascular event – acute myocardial infarction (AMI).

**Methods:**

Consecutive patients with suspected OSA were enrolled and underwent nocturnal respiratory study and electrocardiography monitoring. The minimal oxygen saturation (minSpO_2_) was determined from the oxygen saturation curve under a subject-specific search window. Primary HR metrics such as maximal HR in response to minSpO_2_ and respiratory event-specific HR variability were computed from the synchronized recordings. Multivariate regression analyses were conducted to analyze the associations between individualized HR metrics and the occurrence of AMI.

**Results:**

Of 2,748 patients recruited, 39% (*n* = 1,071) had moderate-to-severe OSA (respiratory event index, REI ≥ 15), and 11.4% (*n* = 313) patients had AMI. Patients with AMI experienced severe OSA, severe minSpO_2_, and greater HR reactions. Patients with minSpO_2_ <90% had an adjusted odds ratio (OR) of 1.48 [95% confidence interval (CI): 1.09–2.00, *p* = 0.012) for AMI. Notably, minSpO_2_-induced elevated mean HR response (HR_mean_ > 73 bpm) was significantly associated with AMI (OR 1.72, 95% CI: 1.32–2.23, *p* < 0.001). Patients with both severe minSpO_2_ (<90%) and elevated HR_mean_ carried an additive OR of 2.65 (95% CI: 1.74–4.05, *p* < 0.001) for the risk of AMI after adjustment for potential confounders. A large total power spectrum specific to respiratory events was correlated with an adjusted OR of 0.61 for AMI risk.

**Conclusion:**

Patients with substantial HR reactions to OSA-induced oxygen nadir and restricted cardiac cycle shifting to respiratory events were likely at increased risk of developing AMI. Detection of nocturnal HR response to hypoxemia may help improve cardiovascular risk stratification.

## Introduction

The direct association with an augmented risk of cardiovascular (CV) morbidity and mortality has raised obstructive sleep apnea (OSA), a pervasive sleep-breathing disorder, as a critical public health problem (1, 2). Acute myocardial infarction (AMI), accounting for approximately 1.8 million deaths annually, remains the leading cause of CV morbidity and mortality worldwide (3). Previous clinic-based cohorts suggested that OSA is an independent risk factor for AMI (1, 4, 5). However, in a recent multi-center randomized study, moderate-to-severe OSA did not appear to impose an additional CV risk (6). Besides, OSA therapy with continuous positive airway pressure (CPAP) in recent randomized clinical trials (6–8) has failed to reduce CV events or mortality. These neutral findings may be partly related to the metrics used to evaluate OSA, and notably, the heterogeneity of CV risk across different phenotypes.

The conventional OSA severity measures simply focus on the frequency of apnea or hypopnea interruptions, while neglecting their immediate hypoxemic and autonomic consequences. A previous large-scale study found that minimal oxygen saturation (minSpO_2_) <78%, but not apnea–hypopnea index (AHI), was independently correlated with an increased risk of sudden cardiac death (9). In patients with coronary artery disease, we reported that minSpO_2_ was independently associated with left ventricular hypertrophy, a marker of CV risk (10). Hypoxemia in OSA is thus postulated as a primary factor preceding subclinical or overt CV damage (11). Besides, cyclical hypoxemia could also elicit a high cardiac variability or sympathetic activity, such as heart rate (HR) accelerations, and contribute to fatal CV events during sleep (1, 12). In this regard, it is plausible that a surge in HR to minSpO_2_ or respiratory events could encapsulate the enormous sympathetic reactivity of OSA and may be one of the mediators of increased CV risk (13, 14).

However, to the best of our knowledge, no prior studies have addressed the HR response measures to minSpO_2_ and heart rate variability (HRV) specific to respiratory events due to OSA and their effects on the occurrence of AMI. Therefore, in the present study, we sought to explore how minSpO_2_-specific HR reactions and HRV parameters identify groups of individuals at particular risk of AMI in a large population with potential CV risk.

## Materials and Methods

### Study Population

Consecutive patients with suspected sleep apnea between 2015 and 2020 at the Guangdong Cardiovascular Institute were enrolled. Patients were considered with suspected sleep apnea if they have the following characteristics: (1) risk factors, including overweight or obese, male gender, older age, postmenopausal state in women, enlarged upper airway soft tissues (e.g., tonsils, adenoids, and tongue), and craniofacial abnormalities (e.g., retrognathia and micrognathia), and (2) clinical symptoms and signs, including excessive sleepiness, fatigue, or unrefreshing sleep, snoring during sleep, witnessed breathing pauses, choking or gasping during sleep, nocturia, nocturnal gastroesophageal reflux, and morning headache. Patients who successfully underwent a cardiorespiratory sleep study and have not been previously diagnosed with OSA were included. Participants who met the following criteria were excluded: previous diagnosed OSA and received treatment with CPAP or other modalities; atrial fibrillation or other significant arrhythmias; and had implanted pacemakers or defibrillators. A total of 2,989 patients were screened for suspected sleep apnea by obtaining the above risk factors and clinical features *via* a detailed medical history taking and careful physical examination, as well as Epworth Sleepiness Scale (ESS) quantification. Among them, 151 patients with atrial fibrillation, nine patients with pacemakers or defibrillators, and six patients with previously diagnosed OSA were excluded. Of the remaining 2,823 patients who underwent nocturnal respiratory study and simultaneous electrocardiogram (ECG) recording, 75 patients whose monitoring data were of insufficient quality, which made proper interpretation difficult, were excluded. In total, 2,748 eligible patients were included in the final data analysis. The study was approved by the Clinical Ethics Committee of Guangdong Provincial People’s Hospital (No. GDREC2018567), and written informed consent was obtained from all participants.

### Clinical Data Acquisition

Demographics, anthropometrics, previous medical history, medications, and daytime ESS score were collected on admission. Blood samples were obtained immediately after admission using EDTA-containing tubes. The baseline estimated glomerular filtration rate (eGFR) was calculated using the Cockcroft–Gault formula. Fasting blood samples were taken in the morning after admission to determine fasting glucose and serum lipids. Two-dimensional transthoracic echocardiography was performed to evaluate the left ventricular ejection fraction. The medical diagnoses were retrieved from the electronic recording system. The diagnosis of AMI was based on clinical manifestations, ECG findings, myocardial enzymes, echocardiography, and coronary angiography according to the criteria proposed by the Third Universal Definition of Myocardial Infarction (15). The diagnosis of AMI and OSA were confirmed at the same time when patients were admitted and hospitalized during the study period.

### Nocturnal Respiratory Study

All eligible patients with suspected sleep apnea underwent Type III nocturnal respiratory monitoring (Alice PDx, Philips 6 Investment Co., Shanghai, China) during the first 24–72 h after admission to evaluate the presence and severity of OSA. As for those with the presence of AMI, these patients were being admitted due to AMI and were evaluated for OSA within the same hospitalization period. Airflow was measured with a nasal pressure cannula, while respiratory efforts were measured utilizing respiratory inductance plethysmography. Apnea and hypopnea events were determined according to the criteria of the American Academy of Sleep Medicine (16). Apnea was defined as a ≥90% reduction of inspiratory airflow for ≥10 s while hypopnea was defined as ≥30% reduction in airflow associated with ≥3% desaturation for ≥10 s. The respiratory event index (REI) was calculated as the number of apnea and hypopnea episodes per recorded hour. The oxygen desaturation index (i.e., the number of ≥3% oxygen desaturations per recorded sleeping hour) was also recorded. A cut-off REI ≥5 events/h was used to define the existence of OSA. Patients with OSA were classified into mild (5 ≤ REI < 15), moderate (15 ≤ REI < 30), and severe (REI ≥ 30) groups.

Nasal airflow and pulse oximetry were used to assess the severity of hypoxemic sequelae of OSA. The key indices analyzed in our study included: total duration of 3% oxygen desaturation, total duration of respiratory events, minSpO_2_, mean SpO_2_ levels, time spent with SpO_2_ <90%, and nocturnal hypoxic burden (only ≥3% oxygen deoxygenation events considered) (10, 17, 18). All recordings were scored manually or calculated by an experienced sleep technician blinded to the clinical data.

### Determination of Heart Rate Response to Minimal Oxygen Saturation and Respiratory Event

A single-lead ECG recording device (Shanghai YueGuang Technology Co. Ltd.) was attached to the patient’s body simultaneously with the Type III portable monitoring device. The ECG signals were manually inspected, whereas noisy segments and other artifacts were excluded from the analysis due to the patient’s movement. At every second, the HR was calculated from the R–R intervals of the raw ECG signals and was retro-graphed to configure an HR tracing curve with a wholly synchronized timescale with Type III portable monitoring channels. For each individually identified apnea or hypopnea, the SpO_2_ signals were traced before and after the end of the obstructive respiratory event until two SpO_2_ peaks on each side were identified. The average oxygen desaturation curve for each participant was determined by overlaying SpO_2_ signals with respect to the end of events. This criterion yielded a search window for the determination of individual oxygen desaturation curves (17). Our target interest was the search window in which the nadir oxygen desaturation, that is, minSpO_2_ throughout the night was located, and the corresponding HR curve was determined.

**FIGURE 1:**
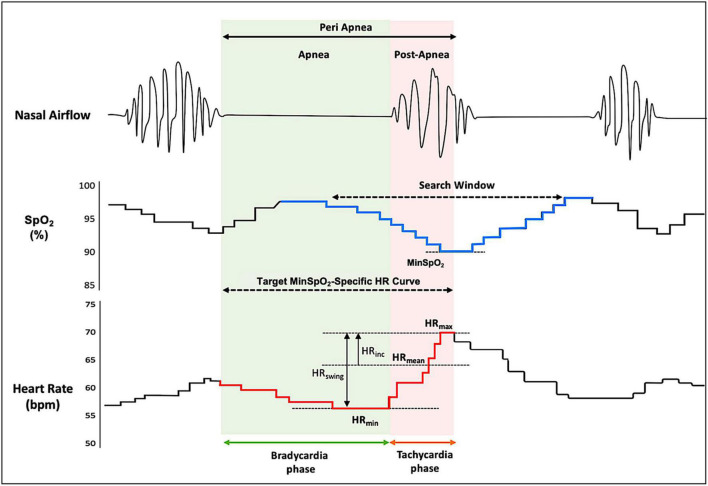
Typical heart rate response during a sleep apneic event. The figure demonstrates a typical pattern of HR tracings (redline, lower) during the peri-apnea phase, with a synchronized timescale illustrating oxygen saturation (SpO_2_) curve (blue line, middle) and nasal airflow (upper). The search window was yielded from overlaying respiratory event–associated oxygen desaturation curve (see section “Materials and Methods” for details). The minSpO_2_ is identified based on subject-specific search window, and a corresponding target HR response curve is determined. HR, heart rate; HR_min_, minimal HR during apnea phase that elicits a subsequent minSpO_2_; HR_max_, maximal HR in response to minSpO_2_ during post-apnea phase; HR_mean_, mean HR representing the average of the HR_max_ and the HR_min_; HR_swing_, HR swing indicative of the difference between HR_max_ and HR_min_; HR_inc_, the increment of HR from the HR_mean_ during the peri-apneic period; SpO_2_, oxygen saturation.

The primary minSpO_2_-specific HR metrics during the peri-apneic period included HR_min_, minimal HR during apnea phase that elicits a subsequent minSpO_2_; HR_max_, maximal HR in response to minSpO_2_ during post-apnea phase; HR_mean_, mean HR representing the average of the HR_max_ and the HR_min_; HR_swing_, HR_swing_ indicative of the difference between HR_max_ and HR_min_; and HR_inc_, the increment of HR from the HR_mean_ during the peri-apneic period (Figure 1). HRV measures specific to minSpO_2_, and respiratory events were also computed based on R–R intervals. Fourier transformation was applied to calculate the frequency domains, among which low-frequency (LF) and high-frequency (HF) ratios were used to quantify the vago-sympathetic balance.

### Statistical Analysis

Continuous variables were described as the mean ± standard deviation, and one-way ANOVA was used to compare differences between groups. The unpaired *t*-test was used for normally distributed variables, while the Mann–Whitney *U* test was used for continuous variables that were not normally distributed. Categorical variables were described by proportions, and Pearson’s Chi-square test or Fisher’s exact test was used to compare the differences between groups as appropriate. To determine the risk factors for the occurrence of AMI, both univariate and multivariate logistic regression analyses were conducted. Confounders included in the multivariate models were the ones that are correlated with AMI and clinically relevant factors. To assess the association between the minSpO_2_-specific HR metrics and the composite variables of HR metrics and minSpO_2_, and AMI prevalence, logistic regression analyses were applied to calculate the odds ratio (OR) and its associated 95% confidence intervals (CIs). Given the novelty of the minSpO_2_–HR metrics, and uncertainty over the associations’ linearity, preliminary associations between various quartiles and AMI were facilitated to determine the model structure and thresholds for individual HR metrics. A two-sided value of *p* < 0.05 was considered as statistically significant. Statistical analyses were performed using the R statistical software (R.3.6).

## Results

### Baseline Characteristics

Of 2,748 studied participants (77.8% male, aged 57.0 ± 11.7 years), 699 (25%) had normal REI, whereas 978 (36%) had mild, 589 (21%) had moderate, and 482 (18%) had severe OSA quantified by REI, respectively. Most participants did not report excessive daytime sleepiness as assessed by the ESS Score. There was a high comorbidity of hypertension (58.9%), diabetes mellitus (24.7%), and dyslipidemia (15%) across populations with different severity of OSA. The prevalence of coronary artery disease reached approximately 59% (*n* = 1,623), whereas AMI accounted for 11.4% (*n* = 313) (Table 1). Moreover, patients with severe OSA quantified by REI, were heavier, male-predominant, had a higher prevalence of AMI, diabetes mellitus, hypertension, and had higher levels of triglycerides, lower eGFR, and lower ejection fraction (Table 1).

**TABLE 1:** Baseline characteristics.

Variables	Non-OSA *n* = 699	Mild OSA *n* = 978	Moderate OSA *n* = 589	Severe OSA *n* = 482	All *n* = 2,748	*p*-Value
Age, year	54.4 ± 13.0	58.1 ± 10.8	58.6 ± 10.8	56.7 ± 11.7	57.0 ± 11.7	<0.001
Male, *n* (%)	481 (68.8)	742 (75.9)	492 (83.5)	424 (88.0)	2,139 (77.8)	<0.001
BMI, kg/m^2^	24.2 ± 3.1	25.3 ± 3.1	26.4 ± 3.2	27.3 ± 4.0	25.6 ± 3.5	<0.001
Diabetes mellitus, *n* (%)	133 (19.0)	230 (23.5)	173 (29.4)	144 (29.9)	680 (24.7)	<0.001
Dyslipidemia, *n* (%)	99 (14.2)	153 (15.6)	89 (15.1)	70 (14.5)	411 (15.0)	0.851
Hypertension, *n* (%)	339 (48.5)	568 (58.1)	371 (63.0)	341 (70.7)	1,619 (58.9)	<0.001
CAD, *n* (%)	372 (53.2)	602 (61.6)	371 (63.0)	278 (57.7)	1,623 (59.1)	<0.001
AMI, *n* (%)	65 (9.3)	115 (11.8)	73 (12.4)	60 (12.4)	313 (11.4)	<0.001
Total cholesterol, mmol/L	4.4 ± 1.1	4.5 ± 1.2	4.5 ± 1.3	4.6 ± 1.4	4.5 ± 1.2	0.440
LDL-C, mmol/L	2.9 ± 0.9	2.9 ± 0.9	3.0 ± 0.9	3.0 ± 0.9	2.9 ± 0.9	0.278
Triglycerides, mmol/L	1.6 ± 1.5	1.9 ± 1.9	1.8 ± 1.5	2.1 ± 2.3	1.8 ± 1.8	<0.001
FBG, mmol/L	5.1 ± 1.6	5.5 ± 2.0	5.7 ± 2.4	5.7 ± 2.2	5.5 ± 2.1	0.011
Creatinine, μmol/L	83.5 ± 58.5	87.2 ± 56.0	94.3 ± 79.2	109.4 ± 105.0	91.7 ± 73.1	<0.001
eGFR, ml/min	99.9 ± 29.8	93.8 ± 28.9	90.0 ± 28.0	85.4 ± 31.7	93.1 ± 30.0	<0.001
EF, %	62.3 ± 9.8	61.7 ± 10.0	60.0 ± 11.2	57.5 ± 14.1	60.7 ± 11.1	<0.001
ESS	5.6 ± 3.5	5.8 ± 3.7	6.2 ± 4.1	7.3 ± 4.5	6.1 ± 3.9	<0.001
TRT, h	7.99 (1.11)	8.00 (1.28)	8.05 (1.16)	8.20 (1.29)	8.04 (1.22)	0.013
REI, events/h	2.3 ± 1.2	9.0 ± 2.6	21.6 ± 4.1	43.7 ± 12.7	16.1 ± 15.5	<0.001
3% ODI, events/h	5.9 ± 4.6	14.7 ± 6.9	28.0 ± 8.9	48.8 ± 14.9	21.3 ± 17.2	<0.001
T90, min	1.21 ± 8.2	2.0 ± 7.2	4.8 ± 8.8	13.3 ± 14.3	4.4 ± 10.4	<0.001
Mean SpO_2_, %	95.1 ± 1.6	94.3 ± 1.5	93.9 ± 1.5	92.8 ± 2.2	94.2 ± 1.8	<0.001
MinSpO_2_, %	89.6 ± 2.8	85.9 ± 3.6	81.2 ± 5.6	75.0 ± 9.9	83.9 ± 7.5	<0.001

*Values are given as the mean ± SD.*

*AMI, acute myocardial infarction; BMI, body mass index; CAD, coronary artery disease; eGFR, estimated glomerular filtration rate; EF, ejection fraction; ESS, Epworth Sleepiness Scale; FBG, fasting blood glucose; LDL-C, low-density lipoprotein; minSpO_2_, minimal oxygen saturation; REI, respiratory event index; 3% ODI, 3% oxygen desaturation index; OSA, obstructive sleep apnea; SpO_2_, oxygen saturation recorded by pulse oximetry; TRT, total recording time of sleep; T90, time spent with SpO_2_ lower than 90%.*

### Comparison of Cardiorespiratory Parameters

Patients with AMI had a higher frequency of apneic or hypopneic episodes, that is, greater REI (17.7 vs. 15.9, *p* = 0.046), and greater 3% ODI (23.6 vs. 21.0, *p* = 0.013) compared to those without AMI. There was a significant difference in minSpO_2_ between patients with and without AMI (83.8 vs. 84.7%, *p* = 0.043) among hypoxemic parameters. The resting HR (i.e., the average HR in the period excluding any respiratory events) was at a level of 66.01 ± 11.49 bpm, and no statistical difference was observed between AMI and non-AMI groups (*p* = 0.437). Further analyses on HR response to obstructive apnea followed by a minSpO_2_, HR_max_, HR_mean_, and HR_min_ appeared significantly greater than resting HR and greater in patients with AMI (Table 2). No significant difference in other HR metrics, including HR_swing_, HR_inc_, and HR_*dur*_ was found between AMI groups.

**TABLE 2:** Sleep respiratory parameters and minSpO_2_-specific HR metrics among patients with and without AMI.

Variables	No AMI	AMI	All	*p*-Value
REI, events/h	15.915.6	17.7 ± 15.4	16.1 ± 15.5	0.046
3% ODI, events/h	21.0 ± 17.1	23.6 ± 17.7	21.3 ± 17.2	0.013
T90, %	4.4 ± 10.5	4.4 ± 9.6	4.4 ± 10.4	0.982
Mean SpO_2_, %	94.2 ± 1.8	94.1 ± 1.7	94.2 ± 1.8	0.333
MinSpO_2_, %	84.7 ± 7.0	83.8 ± 7.5	83.9 ± 7.5	0.043
HR_mean_, bpm	66.8 ± 10.7	70.0 ± 11.5	67.2 ± 10.9	< 0.001
HR_max_, bpm	87.0 ± 20.1	89.1 ± 18.4	87.2 ± 19.9	0.046
HR_min_, bpm	52.1 ± 12.8	54.7 ± 15.5	52.4 ± 13.1	0.001
HR_diff_, bpm	34.8 ± 23.5	34.4 ± 22.4	34.8 ± 23.3	0.772
HR_inc_, bpm	20.2 ± 15.9	19.1 ± 14.6	20.1 ± 15.8	0.282

*AMI, acute myocardial infarction; CI, confidence interval; HR, heart rate; HR_min_, minimal HR during apnea phase that elicits a subsequent minSpO_2_; HR_max_, maximal HR in response to minSpO_2_ during post-apnea phase; HR_mean_, mean HR representing the average of the HR_max_ and the HR_min_; HR_swing_, HR swing indicative of the difference between HR_max_ and HR_min_; HR_inc_, the increment of HR from the HR_mean_ during the peri-apneic period; minSpO_2_, minimal oxygen saturation; OR, odds ratio; REI, respiratory event index; 3% ODI, 3% oxygen desaturation index; OSA, obstructive sleep apnea; SpO_2_, oxygen saturation recorded by pulse oximetry; T90, time spent with SpO_2_ lower than 90%.*

### Associations Between Minimal Oxygen Saturation-Specific Heart Rate Response and Acute Myocardial Infarction

Among various hypoxemic measures, minSpO_2_ remained significantly associated with AMI risk after adjustment for potential confounders (Supplementary Table 1). The severity of minSpO_2_ and the minSpO_2_-specific HR_max_, HR_mean_, and HR_min_ were divided into two distinct groups, respectively, based on the preliminary logistic regression results (Supplementary Tables 1, 2). As shown in Table 3, patients with minSpO_2_ <90% had an OR of 1.48 (95% CI: 1.09–2.00, *p* = 0.012) for AMI risk after adjusting for possible confounders including age, gender, body mass index (BMI), hypertension, dyslipidemia, eGFR, and diabetes mellitus. Notably, substantial HR reactions to minSpO_2_ were significantly associated with an increased likelihood of AMI (HR_mean_ > 73 bpm, OR 1.72, 95% CI: 1.32–2.23; HR_max_ > 83 bpm, OR: 1.31, 95% CI: 1.03–1.67; and HR_min_ > 60 bpm, OR: 1.94, 95% CI: 1.49–2.52; Table 3) even after adjusting for confounders.

**TABLE 3:** Relationship between minSpO_2_ and minSpO_2_-specific HR metrics with AMI.

	Unadjusted		Model 1		Model 2	
Items	OR (95% CI)	*p*-Value	OR (95% CI)	*p*-Value	OR (95% CI)	*p*-Value
MinSpO_2_, <90%	1.47 (1.10−1.96)	0.009	1.47 (1.08−1.98)	0.014	1.48 (1.09−2.00)	0.012
HR_mean_, >73 bpm	1.78 (1.38−2.29)	<0.001	1.78 (1.37−2.30)	<0.001	1.72 (1.32−2.23)	<0.001
HR_max_, >83 bpm	1.36 (1.08−1.73)	0.011	1.34 (1.06−1.70)	0.016	1.31 (1.03−1.67)	0.028
HR_min_, >60 bpm	1.98 (1.53−2.53)	<0.001	2.02 (1.56−2.61)	<0.001	1.94 (1.49−2.52)	0.001

We further examined whether associations between significant HR response and AMI risk would be more robust in patients with severe hypoxemia quantified by minSpO_2_. The mixed groups with various magnitudes of HR metrics and minSpO_2_ were analyzed to evaluate the synergistic effects on the estimated risk for AMI. Patients with severe minSpO_2_ (i.e., <90%), if an elevated HR response was accompanied, would be at a ≥2-fold higher risk for developing AMI. For instance, patients with minSpO_2_ <90% and an elevated minSpO_2_-related HR_mean_ > 73 bpm had 2.65 times (95% CI: 1.74–4.05, *p* < 0.001) increased risk of AMI than those with minSpO_2_ ≥90% and mild HR response (HR_mean_ ≤ 73 bpm), as shown in Table 4.

**TABLE 4:** Associations of composite metrics of minSpO_2_ and minSpO_2_-specific HR metrics with AMI.

	Unadjusted		Model 1		Model 2	
Items	OR (95% CI)	*p*-Value	OR (95% CI)	*p*-Value	OR (95% CI)	*p*-Value
**MinSpO_2_ + HR_mean_**						
Group 1	1.00		1.00		1.00	
Group 2	1.41 (0.83−2.39)	0.210	1.42 (0.83−2.43)	0.196	1.39 (0.81−2.37)	0.234
Group 3	1.35 (0.93−1.95)	0.114	1.36 (0.93−1.99)	0.113	1.38 (0.94−2.01)	0.103
Group 4	2.74 (1.82−4.13)	<0.001	2.74 (1.80−4.18)	<0.001	2.65 (1.74−4.05)	<0.001
**MinSpO_2_ + HR_max_**						
Group 1	1.00		1.00		1.00	
Group 2	1.55 (0.90−2.67)	0.114	1.53 (0.88−2.64)	0.130	1.54 (0.89−2.67)	0.124
Group 3	1.66 (1.02−2.68)	0.040	1.65 (1.01−2.68)	0.045	1.70 (1.04−2.77)	0.035
Group 4	2.33 (1.44−3.77)	0.001	2.26 (1.39−3.70)	0.002	2.25 (1.37−3.67)	0.002
**MinSpO_2_ + HR_min_**						
Group 1	1.00		1.00		1.00	
Group 2	1.32 (0.76−2.31)	0.329	1.40 (0.80−2.46)	0.239	1.35 (0.77−2.37)	0.304
Group 3	1.24 (0.87−1.77)	0.228	1.27 (0.88−1.83)	0.196	1.28 (0.89−1.85)	0.184
Group 4	2.79 (1.89−4.11)	<0.001	2.91 (1.94−4.36)	<0.001	2.81 (1.87−4.21)	<0.001

*MinSpO_2_ and HR metrics (including HR_max_, HR_mean_, and HR_min_) yield a total of four independent groups for individual HR response metric. Group 1 (Control) represents participants with minSpO_2_ ≥90% and mild HR reaction (i.e., HR_mean_ ≤ 73 bpm, HR_max_ ≤ 83 bpm, or HR_min_ ≤ 60 bpm). Group 2 represents participants with minSpO_2_ <90% and mild HR reaction. Group 3 represents participants with minSpO_2_ ≥90% and substantial HR reaction (i.e., HR_max_ > 83 bpm, or HR_mean_ > 73 bpm, or HR_min_ > 60 bpm). Group 4 represents participants with minSpO_2_ <90% and substantial HR reaction. Each line represents a separate regression model. Model 1 adjusted for age, gender, and BMI. Model 2 adjusted for age, gender, BMI, hypertension, dyslipidemia, eGFR, and diabetes mellitus.*

*AMI, acute myocardial infarction; BMI, body mass index; CI, confidence interval; eGFR, estimated glomerular filtration rate; HR, heart rate; minSpO_2_, minimal oxygen saturation. HR_min_, minimal HR during apnea phase that elicits a subsequent minSpO_2_; HR_max_, maximal HR in response to minSpO_2_ during post-apnea phase; HR_mean_, mean HR representing the average of the HR_max_ and the HR_min_; OR, odds ratio.*

### Associations Between Sleep Apnea-Associated Heart Rate Variability Parameters and Acute Myocardial Infarction

HRV parameters were divided into quartiles and the first group was considered the reference group. Among minSpO_2_ variables, the mean NN interval and LF spectral band showed a protective role in AMI risk (Table 5). The non-respiratory event-LF/HF was slightly higher in patients with AMI and severe OSA, than in non-AMI patients (0.80 vs. 0.77) and non-OSA patients (0.80 vs. 0.60), respectively. Patients with a higher LF/HF ratio would be at increased risk of AMI (OR = 1.34) after adjusting for age, gender, and BMI. Notably, respiratory event-associated total power (TP) spectral band, NN, and standard deviation of NN interval (SDNN) were related to decreased ORs of AMI incidence.

**TABLE 5:** Significant sleep apnea-specific heart rate variability parameters associated with AMI risk.

HRV variables*	Unadjusted		Model 1		Model 2	
	OR (95% CI)	*p*-Value	OR (95% CI)	*p*-Value	OR (95% CI)	*p*-Value
**minSpO_2_-related**						
LF > 77.2	0.69 (0.49−0.96)	0.028	0.62 (0.45−0.88)	0.007	0.64 (0.45−0.90)	0.011
NN > 1.02	0.50 (0.36−0.70)	<0.001	0.48 (0.34−0.67)	<0.001	0.49 (0.34−0.69)	<0.001
**RE-related**						
TP > 1570	0.62 (0.45−0.87)	0.006	0.57 (0.41−0.80)	0.002	0.61 (0.43−0.86)	0.005
NN > 1.02	0.49 (0.35−0.68)	<0.001	0.46 (0.33−0.64)	<0.001	0.49 (0.35−0.68)	<0.001
SDNN > 0.101	0.68 (0.49−0.96)	0.029	0.64 (0.45−0.90)	0.012	0.69 (0.48−0.98)	0.041
**NRE-related**						
LF/HF > 0.91	1.63 (1.17−2.27)	0.004	1.34 (0.95−1.88)	0.098	1.36 (0.96−1.92)	0.084

*The fourth quartiles were shown with the first quartile as the referenced group. Model 1 adjusted for age, gender, and BMI. Model 2 adjusted for age, gender, BMI, hypertension, dyslipidemia, eGFR, and diabetes mellitus.

*OR, odds ratio; minSpO_2_, minimal oxygen saturation; RE, respiratory event; NRE, non-respiratory events; TP, total power; SDNN, standard deviation of NN interval; LF, low-frequency; HRV, heart rate variability.*

## Discussion

The present study provides new insights into the quantification of HR response due to OSA and its capacity for AMI risk stratification. The novel and principal findings are that the hypoxemia measure—minSpO_2_ that characterizes OSA-related hypoxemic severity correlated with incident AMI. Notably, patients with pronounced minSpO_2_-specific HR reactions (specifically, HR_max_, HR_mean_, and HR_min_) magnified AMI risk by nearly 1.3- to 1.9-fold compared to those with a mild HR response. This association appeared stronger among individuals with severe minSpO_2_ <90%. Analysis of sleep apnea–specific HRV also supported the hidden role of enhanced sympathetic tone and restricted variations in cardiac cycle in the incidental occurrence of AMI.

### The Role of Hypoxemia and Associated Heart Rate Surges

Recent literature has identified novel measures that may accurately quantify respiratory event-specific hypoxemia including the length of obstructive events (19) and the cumulative extent of oxygen desaturation (17, 20), which are postulated as new candidates for OSA-associated CV risk such as CV mortality, all-cause mortality (17, 21), and incident heart failure (22). Nevertheless, the above-mentioned studies paid less attention to the nadir oxygen saturation and its effects on CV risk. In contrast, in our study, the nadir oxygen desaturation, that is, minSpO_2_, appears to be one of the strongest factors that correlate with AMI incidence, beyond REI, 3% ODI, and other hypoxemic measures. Similarly, we reported that minSpO_2_ ≤ 80% could impose a two- to threefold higher odds on the development of left ventricular hypertrophy than those with minSpO_2_ ≥ 90% in patients with coronary artery disease (10). These indicated that minSpO_2_, representing the most severe dip of oxygen saturation, is likely to cause dysfunctional cardiac variability that may predispose patients to CV events. A previous large-scale longitudinal study showed that the minSpO_2_ ≤ 78%, was independently associated with an increased risk of sudden cardiac death (9), suggesting that nadir oxygen desaturation is an essential element precipitating lethal ventricular arrhythmias. In this regard, characterizing minSpO_2_-specific HR changes is an essential avenue to allow more target approaches to preventing CV events.

### Triggering Effect of Periodic Respiratory Event-Specific Heart Rate Response and Variability

An increased sympathetic tone in OSA has been reported to be mostly responsible for the high prevalence of AMI (23) and CV events (1). To detect the rhythmic oscillation in OSA, previous studies used direct recordings of sympathetic fibers by invasive microneurographic techniques and an analysis of HRV (24, 25). Generally, HRV is derived from mathematical analyses of intervals between normal heartbeats, but a few limitations have been observed. The frequency domain analysis of HRV usually requires patients to be stationary and is limited to a fixed cut-point on the overnight graphical tracing of sleep (25). Such an HR measurement approach may be problematic since most OSA patients may not have significant event-free periods during sleep. OSA-induced necessary modifications of breathing patterns may also affect the HR oscillation and could not be distinguished by the HRV analysis (26). Similarly, Shimizu et al. developed a cyclic variation of HR score determined by Holter ECG and concluded that it was a useful screening tool for severe sleep-disordered breathing among patients with heart failure (13). However, the parameters used in that study did not consider the nadir hypoxic effect as minSpO_2_-specific HR metrics did (13). Also, minSpO_2_-specific HR reactions (either maximal, mean, or minimal HR), partly attributed to arousals from sleep (27–29), may reflect the substantive CV reactions to the extreme hypoxic effect.

The non-respiratory event–associated LF/HF in HRV analysis was slightly greater in patients with AMI as well as severe OSA, and as the ratio increased, the AMI risk increased by nearly 36%. This probably supported the link between enhanced sympathetic activity and AMI. In general, standard frequency-oriented OSA measures may serve as the substrate for CV morbidity, while the “hypoxic–sympathetic storm” is more likely to be a critical trigger of CV events. Also, we observed the protective role of a greater TP of spectral bands specific to respiratory events (namely, the square sum of cardiac cycle variations) in AMI risk. In other words, if hypoxemia is accompanied by restricted cardiac cycle shifting or mild HR swing amplitude, it might diminish coronary blood flow and precede individuals to myocardial infarction.

### Proposed Mechanisms Linking Heart Rate Response, Variability, and Acute Myocardial Infarction Risk

The proposed mechanisms linking the minSpO_2_ and specific HR response to it are explained separately for chronic and acute effects on the onset of AMI. Some reports show that OSA exhibits a peak with respect to the onset of CV diseases during sleeping to morning hours (30, 31). Presumably, traditional OSA parameters such as REI, 3% ODI, or hypoxemic duration may exert basal chronic pathophysiologic effects on the long-term development of CV damage. At the same time, the dramatic acute HR surge in response to minSpO_2_ may be an acute trigger factor for AMI. In specific, regarding the chronic effects, long-term intermittent hypoxia and reoxygenation were shown to increase the myocardial infarct size (32). This hypoxemic mechanism is also thought to generate cardiac hyper-excitability by altering sympathovagal balance reflected by cyclical variations in HR (13), and as indicated by an increased basal LF/HF in patients with severe OSA and AMI. In terms of the acute impact, the rapid HR reactions may be associated with accelerated atherogenesis of the coronary artery initiated by endothelial dysfunction through mechanical and metabolic factors (33). If HR reacts dramatically to minSpO_2_, there would be an augmented myocardial demand for oxygen and impaired coronary perfusion due to a shorter diastolic filling duration. The myocardial ischemia will occur, and the infarct size will likely be expanded if accompanied by a high coronary plaque burden (34, 35). A high HR response *per se* has also been associated with coronary plaque disruption (36), rendering patients at a higher risk of suffering from acute AMI.

### Practical Significance

The present study’s findings may suggest the potential association between minSpO_2_-elicited severe HR reactions, variability, and AMI risk in a large population with suspected OSA. This implied that abnormal cardiopulmonary interaction or sympathetic activation might play a potential role in AMI occurrence. The advantages of detecting such biological signals lie in their simplicity of operation, economic cost, non-invasiveness, remote accessibility, and portability. In clinical practice, apart from early recognition of OSA, additional investigation of nocturnal oxygen desaturation, especially minSpO_2_ and HR response may discriminate the vulnerable phenotypes of patients at higher risk of AMI. However, these assumptions need to be further examined by future large-scale studies.

### Study Strengths and Limitations

Our study’s main strengths include first, a large sample size; and second, multi-dimensional analyses of cardiorespiratory measures. Most importantly, we conducted a compressive assessment of the HR metrics associated with minSpO_2_, which has not been widely examined in previous literature. Nevertheless, this study has limitations. First, OSA was detected by portable cardiorespiratory monitoring rather than attended polysomnography (PSG). However, the cost-effective and user-friendly portable device has similar accuracy for OSA assessment compared with PSG (37). It also permits a more stable and actual assessment of the disease status because the first night effects could be largely reduced as fewer connecting devices are required. Second, multivariate models may not grant the complete elimination of the confounding factors for AMI. However, common risk factors for AMI such as dyslipidemia, diabetes mellitus, and hypertension were included in the multivariate models while avoiding overadjustment from other highly collinear variables. Finally, the nature of observational, cross-sectional research would not allow us to determine causality between HR metrics and AMI, which requires future prospective cohort studies.

## Conclusion

In conclusion, increased HR reactions to OSA-induced minSpO_2_ and several HRV indices were independently associated with a higher likelihood of AMI. Detection of nocturnal oxygen saturation and autonomic response to nadir hypoxemia may help improve CV risk stratification. Prospective large-scale randomized studies are warranted to evaluate the prognostic significance of OSA-specific HR metrics on CV outcomes across populations.

## Data Availability Statement

The raw data supporting the conclusions of this article will be made available by the authors, without undue reservation.

## Ethics Statement

The studies involving human participants were reviewed and approved by the Clinical Ethics Committee of Guangdong Provincial People’s Hospital (No. GDREC2018567). The patients/participants provided their written informed consent to participate in this study. Written informed consent was obtained from the individual(s) for the publication of any potentially identifiable images or data included in this article.

## Author Contributions

ZH contributed to the study design, data analysis, data interpretation, and manuscript drafting. YW, KH, and PC contributed to the data acquisition, analysis, and interpretation. JC and LW were responsible for the study design, analysis, interpretation of the data, and critical revision of the manuscript. All authors contributed substantially to the work and agreed to submit the manuscript for publication.

## Conflict of Interest

The authors declare that the research was conducted in the absence of any commercial or financial relationships that could be construed as a potential conflict of interest.

## Publisher’s Note

All claims expressed in this article are solely those of the authors and do not necessarily represent those of their affiliated organizations, or those of the publisher, the editors and the reviewers. Any product that may be evaluated in this article, or claim that may be made by its manufacturer, is not guaranteed or endorsed by the publisher.
