# Supplementary material for: The Nadir Oxygen-Specific Heart Rate Response in Sleep Apnea Links With the Occurrence of Acute Myocardial Infarction
Source: Front Cardiovasc Med. 2022 Apr 26;9:807436. doi: 10.3389/fcvm.2022.807436 (PMC9086507; doi:10.3389/fcvm.2022.807436)
Supplement: Supplementary file 1 [file Data_Sheet_1.docx]

**Supplementary Material**

***Table S1***. Relationship between respiratory parameters and AMI.

|  | **Unadjusted** | | **Model 1** | | **Model 2** | |
| --- | --- | --- | --- | --- | --- | --- |
| **Items** | **OR (95% CI)** | **P value** | **OR (95% CI)** | **P value** | **OR (95% CI)** | **P value** |
| **REI, events/h** |  |  |  |  |  |  |
| REI <5 | 1.00 |  | 1.00 |  | 1.00 |  |
| 5 ≤ REI <15 | 1.30 (0.94- 1.79) | 0.110 | 1.30 (0.93- 1.81) | 0.121 | 1.29 (0.93-1.80) | 0.129 |
| 15 < REI ≤30 | 1.38 (0.97-1.97) | 0.075 | 1.36 (0.93-1.97) | 0.111 | 1.33 (0.91-1.93) | 0.143 |
| REI ≥30 | 1.39 (0.96-2.01) | 0.085 | 1.37 (0.92-2.04) | 0.122 | 1.34 (0.90-1.99) | 0.156 |
| **ODI, events/h** |  |  |  |  |  |  |
| ODI <5 | 1.00 |  | 1.00 |  | 1.00 |  |
| 5 ≤ ODI <15 | 1.39 (0.98-1.98) | 0.069 | 1.42 (0.99-2.04) | 0.057 | 1.41 (0.98-2.03) | 0.063 |
| 15 < ODI ≤30 | 1.31 (0.92-1.87) | 0.140 | 1.35 (0.93-1.96) | 0.110 | 1.33 (0.92-1.93) | 0.134 |
| ***ODI ≥30*** | ***1.73 (1.23-2.43)*** | ***0.002*** | ***1.84 (1.28-2.66)*** | ***0.002*** | ***1.78 (1.23-2.58)*** | ***0.003*** |
| **MinSpO_2_, %** |  |  |  |  |  |  |
| ≥90 | 1.00 |  | 1.00 |  | 1.00 |  |
| ***87-89*** | ***1.48 (1.06-2.06)*** | ***0.021*** | ***1.48 (1.06-2.07)*** | ***0.023*** | ***1.49 (1.07-2.09)*** | ***0.020*** |
| ***81-86*** | ***1.42 (1.00-2.00)*** | ***0.049*** | ***1.41 (0.98-2.01)*** | ***0.043*** | ***1.42 (0.99-2.03)*** | ***0.047*** |
| ***≤80*** | ***1.52 (1.07-2.20)*** | ***0.022*** | ***1.54 (1.04-2.28)*** | ***0.033*** | ***1.56 (1.05-2.32)*** | ***0.028*** |
| **Mean SpO_2_, %** |  |  |  |  |  |  |
| >95 | 1.00 |  | 1.00 |  | 1.00 |  |
| 94-95 | 0.93 (0.68-1.27) | 0.636 | 0.90 (0.65-1.23) | 0.495 | 0.90 (0.65-1.23) | 0.499 |
| 93-94 | 0.86 (0.62-1.18) | 0.338 | 0.79 (0.57-1.09) | 0.154 | 0.78 (0.56-1.09) | 0.151 |
| ≤93 | 0.80 (0.57-1.14) | 0.216 | 0.77 (0.53-1.13) | 0.183 | 0.78 (0.54-1.14) | 0.199 |
| **T90%, min** |  |  |  |  |  |  |
| ≤0.1 | 1.00 |  | 1.00 |  | 1.00 |  |
| 0.1-2.6 | 1.00 (0.73-1.38) | 1.000 | 1.02 (0.73-1.42) | 0.915 | 1.01 (0.73-1.41) | 0.949 |
| 2.6-17.1 | 0.74 (0.53-1.04) | 0.087 | 0.75 (0.52-1.07) | 0.108 | 0.75 (0.52-1.07) | 0.109 |
| >17.1 | 0.91 (0.66-1.26) | 0.566 | 0.93 (0.65-1.33) | 0.696 | 0.93 (0.65-1.32) | 0.671 |
| **NHB, %min/h** |  |  |  |  |  |  |
| ≤7.3 | 1.00 |  | 1.00 |  | 1.00 |  |
| 7.4-18.8 | 1.07 (0.75-1.51) | 0.716 | 1.05 (0.74-1.50) | 0.786 | 1.04 (0.73-1.49) | 0.832 |
| 18.9-50.4 | 1.28 (0.92-1.80) | 0.145 | 1.22 (0.86-1.74) | 0.259 | 1.22 (0.86-1.73) | 0.278 |
| >50.4 | 1.21 (0.87-1.71) | 0.263 | 1.17 (0.81-1.69) | 0.393 | 1.14 (0.79-1.64) | 0.495 |
| **OD duration, min** |  |  |  |  |  |  |
| ≤30.8 | 1.00 |  | 1.00 |  | 1.00 |  |
| 30.9-62.2 | 1.20 (0.86-1.69) | 0.287 | 1.22 (0.86-1.72) | 0.268 | 1.20 (0.85-1.70) | 0.310 |
| 62.3-110.5 | 1.27 (0.91-1.78) | 0.166 | 1.27 (0.89-1.80) | 0.186 | 1.25 (0.88-1.78) | 0.210 |
| >110.5 | 1.13 (0.80-1.60) | 0.479 | 1.12 (0.78-1.61) | 0.539 | 1.09 (0.75-1.56) | 0.663 |
| **RE duration, min** |  |  |  |  |  |  |
| ≤10.0 | 1.00 |  | 1.00 |  | 1.00 |  |
| 10.1-24.3 | 1.08 (0.77-1.53) | 0.653 | 1.08 (0.76-1.54) | 0.662 | 1.09 (0.76-1.56) | 0.636 |
| 24.4-60.3 | 1.25 (0.89-1.75) | 0.196 | 1.20 (0.84-1.70) | 0.313 | 1.19 (0.83-1.69) | 0.341 |
| >60.3 | 1.27 (0.90-1.77) | 0.172 | 1.20 (0.84-1.71) | 0.331 | 1.17 (0.82-1.68) | 0.393 |

Each line represents a separate regression model. REI and ODI analyses were based on conventional classifications. Other variables were divided into quartiles. Model 1 adjusted for age, sex, and BMI. Model 2 adjusted for age, sex, BMI, hypertension, dyslipidemia, eGFR and diabetes mellitus. AMI, acute myocardial infarction; BMI, body mass index; CI, confidence interval; eGFR, estimated glomerular filtration rate; minSpO_2_, minimal oxygen saturation; NHB, nocturnal hypoxic burden; OD, oxygen desaturation typically ≥3%; ODI, 3% oxygen desaturation index; OR, odds ratio; RE, respiratory event; REI, respiratory event index; SpO_2_, oxygen saturation recorded by pulse oximetry.

***Table S2****.* Relationship between metrics of heart rate response to minSpO_2_ and AMI.

|  | **Unadjusted** | | **Model 1** | | **Model 2** | |
| --- | --- | --- | --- | --- | --- | --- |
| **Items** | **OR (95% CI)** | **P value** | **OR (95% CI)** | **P value** | **OR (95% CI)** | **P value** |
| **HR_mean_** |  |  |  |  |  |  |
| Q1 (≤60 bpm) | 1.00 |  | 1.00 |  | 1.00 |  |
| Q2 (61-66 bpm) | 1.11 (0.76-1.62) | 0.578 | 1.14 (0.78-1.66) | 0.514 | 1.11 (0.76-1.63) | 0.577 |
| Q3 (67-73 bpm) | 1.38 (0.96-1.98) | 0.081 | 1.44 (0.99-2.07) | 0.052 | 1.39 (0.96-2.01) | 0.08 |
| ***Q4 (>73 bpm)*** | ***2.07 (1.47-2.90)*** | ***<0.001*** | ***2.11 (1.49-2.98)*** | ***<0.001*** | ***2.00 (1.41-2.84)*** | ***<0.001*** |
| **HR_max_** |  |  |  |  |  |  |
| Q1 (≤74 bpm) | 1.00 |  | 1.00 |  | 1.00 |  |
| Q2 (75-83 bpm) | 1.31 (0.92-1.88) | 0.132 | 1.27 (0.88-1.81) | 0.199 | 1.23 (0.86-1.76) | 0.266 |
| ***Q3 (84-94 bpm)*** | ***1.68 (1.18-2.39)*** | ***0.004*** | ***1.64 (1.15-2.34)*** | ***0.007*** | ***1.56 (1.09-2.23)*** | ***0.016*** |
| ***Q4 (>94 bpm)*** | ***1.50 (1.06-2.14)*** | ***0.023*** | ***1.45 (1.02-2.07)*** | ***0.041*** | ***1.41 (0.99-2.02)*** | ***0.049*** |
| **HR_min_** |  |  |  |  |  |  |
| Q1 (≤46 bpm) | 1.00 |  | 1.00 |  | 1.00 |  |
| Q2 (47-53 bpm) | 0.85 (0.59-1.21) | 0.370 | 0.85 (0.59-1.22) | 0.378 | 0.84 (0.59-1.21) | 0.343 |
| Q3 (54-60 bpm) | 0.94 (0.65-1.34) | 0.726 | 0.97 (0.68-1.40) | 0.885 | 0.94 (0.65-1.36) | 0.752 |
| ***Q4 (>60 bpm)*** | ***1.83 (1.33-2.51)*** | ***<0.001*** | ***1.90 (1.37-2.62)*** | ***<0.001*** | ***1.79 (1.29-2.49)*** | ***0.001*** |
| **HR_swing_** |  |  |  |  |  |  |
| Q1 (≤18 bpm) | 1.00 |  | 1.00 |  | 1.00 |  |
| Q2 (19-27 bpm) | 0.85 (0.60-1.19) | 0.338 | 0.77 (0.54-1.08) | 0.130 | 0.77 (0.55-1.09) | 0.147 |
| Q3 (28-45 bpm) | 0.90 (0.65-1.26) | 0.551 | 0.80 (0.57-1.13) | 0.201 | 0.82 (0.58-1.15) | 0.247 |
| Q4 (>45 bpm) | 1.02 (0.73-1.41) | 0.093 | 0.93 (0.67-1.30) | 0.673 | 0.95 (0.68-1.33) | 0.760 |
| **HR_inc_** |  |  |  |  |  |  |
| Q1 (≤10 bpm) | 1.00 |  | 1.00 |  | 1.00 |  |
| Q2 (11-15 bpm) | 0.87 (0.62-1.21) | 0.409 | 0.80 (0.57-1.13) | 0.203 | 0.82 (0.58-1.14) | 0.237 |
| Q3 (16-23 bpm) | 0.91 (0.65-1.26) | 0.564 | 0.78 (0.56-1.10) | 0.157 | 0.80 (0.57-1.12) | 0.188 |
| Q4 (>23 bpm) | 0.80 (0.57-1.13) | 0.202 | 0.74 (0.53-1.05) | 0.089 | 0.76 (0.54-1.07) | 0.121 |

Each line represents a separate regression model. Individual HR metrics were divided into quartiles. Model 1 adjusted for age, sex and BMI. Model 2 adjusted for age, sex, BMI, hypertension, dyslipidemia, eGFR and diabetes mellitus. AMI, acute myocardial infarction; BMI, body mass index; CI, confidence interval; eGFR, estimated glomerular filtration rate; HR, heart rate; HR_min_, minimal HR during apnea phase that elicits a subsequent minSpO_2_; HR_max_, maximal HR in response to minSpO_2_ during post-apnea phase; HR_mean_, mean HR representing the average of the HR_max_ and the HR_min_; HR_swing_, HR swing indicative of the difference between HR_max_ and HR_min_; HR_inc_, the increment of HR from the HR_mean_ during the peri-apneic period; minSO_2_, minimal oxygen saturation; OR, odds ratio.
